# Effect of temperature on gonadal differentiation and growth of *Leporinus friderici*

**DOI:** 10.1590/1984-3143-AR2023-0158

**Published:** 2024-07-15

**Authors:** Thiago Gonçalves de Souza, Mariana Roza de Abreu, Rafael Yutaka Kuradomi, Sergio Ricardo Batlouni

**Affiliations:** 1 Centro de Aquicultura, Universidade Estadual Paulista, Jaboticabal, SP, Brasil; 2 Instituto de Ciências Exatas e Tecnologia, Universidade Federal do Amazonas, Itacoatiara, AM, Brasil

**Keywords:** sex determination, gonads, females, fish, wild

## Abstract

This study aimed to investigate the effect of temperature on gonadal differentiation, growth, survival, and sex ratio of *Leporinus friderici* reared at 25 °C or 29 °C from 50 to 240 days after eclosion (DAE) in a water recirculation system. A total of 110 fish at 50 DAE (6.7 ± 0.1 cm and 6.1 ± 0.3 g) were equally and randomly distributed in 10 boxes (90 L) (11 fish/box, 5 boxes/temperature). One fish from each experimental unit was randomly sampled at 50, 70, 90, 110, 130, 150, 170, 190, 210 and 240 DAE. Female gonadal differentiation started at 150 DAE (11.4 ± 0.0 cm and 16.4 ± 0.0 g) at 25 °C and at 170 DAE (10.7 ± 0.7 cm and 27.7 ± 8.5 g) at 29 ºC, while testes differentiation only occurred at 29 °C from 190 DAE (12.1 ± 0.0 cm and 38.0 ± 0.0 g). Of 50 fishes sampled in each condition, 17 (12 females and five males) and three (three females) displayed gonadal differentiation at 29 °C and 25 °C, respectively. Final biometric values at 29 °C were twice those obtained at 25 °C, reaching 13.9 ± 0.65 cm and 57.3 ± 10.12 g *versus* 11.2 ± 0.39 cm and 28.5 ± 2.95 g, respectively. While temperature clearly influenced gonadal differentiation and growth, it had inconclusive effects on sex ratio. The higher temperature (29 °C) has direct implications for the production of this species, as it accelerates growth without causing mortality.

## Introduction

*Leporinus friderici* (Bloch, 1794) (Characiformes, Anostomidae) is a gonochoristic rheophilic species (Agostinho et al., 2007) that exhibits external fertilization, with a reproductive period occurring generally from September through April in South America (Lopes et al., 2000). This species belongs to the genus *Leporinus* (Agassiz, 1829), which comprises at least four species listed among the fish species produced in Brazil with a production of 2,806 tons in 2021, according to official data from Instituto Brasileiro de Geografia e Estatística (IBGE) (IBGE, 2021). *L. friderici* is also of great importance for subsistence fishing, being prominently found in various rivers and reservoirs in South America (Lopes et al., 2000; Albrecht and Caramaschi, 2003; Nascimento et al., 2023). Moreover, *L. friderici* is among the principal fish species landed at the fishing ports monitored on the lower Amazon (Furtado et al., 2023) and is also registered in fishing landing in Peru where it is also one of the omnivorous captured fish species used for consumption of the Peruvian amazon (García-Dávila et al., 2018). However, due to its migratory behavior, which involves long-distance movements during the reproductive period, this species may be negatively affected by the construction of dams for hydroelectric power plants (Durães et al., 2001; Albrecht and Caramaschi, 2003; Agostinho et al., 2007), leading to reproductive impairment and subsequent decline in wild stocks. To address this issue, juveniles of *L. friderici* are hatchery-reared and sold to hydroelectric power plants companies for restocking rivers impacted by dam construction (Valenti et al., 2021). Therefore, there is a significant demand for *L. friderici* fingerlings for aquaculture and restocking programs across a large part of Brazil (Souza et al., 2020; Valenti et al., 2021; Vidal and Batlouni, 2023).

In the wild, females of *L. friderici* generally reach larger sizes than males (Rêgo et al., 2008), suggesting that monosex female rearing could potentially enhance production in captivity, although it has not been commercially implemented to date. Indeed, monosex populations are commonly utilized in other fish production systems, as they promote the development of the gender that provides certain zootechnical advantage, typically favoring the one with the superior growth rates (Singh, 2013). In fish, this is possible because both genetic and external interferences (such as temperature, social factors and the use of agents such as hormones and enzymes) can interfere in the process of sex differentiation (Devlin and Nagahama, 2002; Fernandino and Hattori, 2019). External influences, such as exposure to specific temperatures for certain periods and/or the application of hormonal therapies, can alter the fate of germ and somatic cells in the gonads, thereby influencing the sex determination of fish.

In fish, genetic mechanisms of sex determination involve polygenic or monogenic systems, with factors located in both autosomes and sex chromosomes. In the case of sex chromosomes, male (XY) and female (ZW) heterogametic systems can occur (Devlin and Nagahama, 2002). Regarding the genus *Leporinus*, which encompasses approximately 81 species (Nascimento et al., 2023), the majority do not possess sex chromosomes. However, seven species, not including *L. frederici*, have been described as having the ZW heteromorphic system, where the “W” chromosome is primarily composed of a large accumulation of microsatellites (Poltronieri et al., 2014). *L. friderici* is one of them for which there is no evidence of the presence of sex chromosomes (Marreta et al., 2012).

Among the various methods of influencing in sex determination, one of the most employed is the modulation of water temperature. In temperature-dependent sex determination, temperature affects gonadal steroidogenesis mainly by modulating the expression of the *cyp19a1a* gene (Strüssmann and Nakamura, 2002). This gene encodes a member of the cytochrome P450 superfamily, which plays a key role in the conversion of androgens to estrogens (Simpson et al., 1994). Steroid hormones play a crucial role in regulating the process of sexual differentiation in teleost fishes (Baroiller and D’Cotta, 2001). Overall, the androgen 11-ketotestosterone (11-KT) primarily regulates testicular development, while 17β-estradiol (E_2_) induces and maintains ovarian development (Devlin and Nagahama, 2002). An excess of 11-KT induces male differentiation, whereas an excess of E_2_ induces female differentiation in several species (Rougeot et al., 2007).

In this context, despite the abundant fish biodiversity in South America, knowledge regarding the sex determination mechanisms of these species and efforts to produce monosex populations are scarce. Most studies on sexual inversion are temperature-dependent and focused on species of the Atheriniformes group, which have successfully achieved population of 100% males through exposure to low temperatures, as observed in the kingfish, *Odontesthes bonariensis* (Fernandino and Hattori, 2019). Regarding Neotropical farmed species, to the best of our knowledge, only *Astyanax altiparanae* (Bem et al., 2012), *Rhamdia quelen* (Amaral-Junior et al., 2008) and *Leporinus macrocephalus* (Pereira et al., 2020) have been subjected to hormone-induced sex inversion.

Therefore, considering that the production of monosex female populations may enhance species productivity, the objective of this study was to determine the onset of gonadal differentiation of *L. friderici* exposed to temperatures experienced by the species in the wild during pre-spawning (winter months) (25 °C) and at spawning season (summer months) (29 ºC) (Brito et al., 1999) from 50 to 240 days after eclosion (DAE), and to evaluate the influence of these temperatures on standard length (SL), body mass (BM), survival and sex ratio.

## Methods

This study was conducted in compliance with National Council for the Control of Animal Experimentation (CONCEA) and approved by the Animal Ethics and Welfare Committee (CEUA) from UNESP, Jaboticabal, SP, Brazil, under permission number 019375/13 for being in accordance with ethical principles in animal experimentation.

### Origin of the animals

The experiment was carried out at the Centro de Aquicultura da Universidade do Estado de São Paulo (CAUNESP), Jaboticabal, São Paulo, Brazil (21°15′17” S, 48°19′20” W) between February and August. After August the characteristics of the winter months (dry and sometimes cold) in the Jaboticabal (Rolim and Aparecido, 2015) region intensified and it is no longer possible to handle fish. It is expected that fish have already been transferred to growth out phase conditions before August.

The fingerlings used in the study were obtained from induced spawning in captivity at CAUNESP. To this end, we used a protocol of carp pituitary extract with a relatively lower dose than usual for Neotropical species (0.5 mg/kg and 1.0 mg/kg – six hours apart), which has previously been shown to be the best for obtaining viable embryos in this species (Souza et al., 2020). The breeders used for spawning were captured from the Sapucai Mirim River in São Joaquim da Barra, São Paulo, Brazil. These breeders had been previously kept in captivity for a period of two years before being used for breeding (Souza et al., 2020).

### Reproduction and larviculture (pre-experimental period)

After spawning in a semi-natural system, approximately 2,000 eggs (pool of gametes of eight females and eight males) were transferred to 120 L conical funnel-type incubator (*n* = 6). After hatching, larvae were kept for a period of 15 days in the incubators, fed four times a day, as follows: 1st to 4th days: 50 Artemia nauplii / larva / day (BioArtemia. Ltda, RN, Brazil.); 5th to 8th days: 100 Artemia nauplii / larva / day; 9th to 10th days: 150 Artemia nauplii / larva / day; 10th to 15th days: 300 Artemia nauplii / larva / day. Additionally, powdered commercial feed (with 40% crude protein) was provided *ad libitum*, along with boiled chicken egg, which was given twice a day. The boiled chicken egg was prepared by boiling a whole egg in water and then rapidly cooling it with cold water. This type of food was supplemented due to the relatively small size of the mouth of *L. friderici*.

On the 16th day after eclosion (DAE), the larvae were transferred to 200 m^3^ earthen ponds (~100 larvae/m^2^ at CAUNESP, where they were kept until reaching 50 DAE. The ponds were fertilized and managed specifically for larviculture of Neotropical native species (Graeff et al., 2008). The larvae were fed *ad libitum*, twice a day, with mashed commercial food for omnivorous fish, which had a composition of 55% crude protein, 10% moisture content, 7% ethereal extract, 2.8% fiber, 4.2% calcium, and 1.5% phosphorus.

### Experimental design

A total of 110 individuals at 50 DAE were used. These individuals had a mean standard length (SL) of 6.7 ± 0.1 cm and a mean body mass (BM) of 6.1 ± 0.3 g. The fish were equally and randomly distributed into 10 boxes (five boxes per condition), with 11 fish per box. The resulting density of fish in each box was approximately 0.75 g/L. The choice to start at 50 DAE onwards was due to the following reasons: a) we had already conducted pilot experiments and we knew that differentiation did not occur before; b) the fish are more fragile before 50 days, and we could perish them due to management and the support capacity of the recirculation system.

The experimental conditions consisted of two independent water recirculation systems, each equipped with biological filters and heat pumps with digital controllers. The heat pumps allowed for the maintenance of a constant temperature, which was monitored daily through a digital panel. One of the systems was maintained at 25 °C using a heat exchanger adapted for heating/cooling (Nautilus AA-45 Aquahot Automatic – adapted), while the other system was maintained at 29 °C using a heat exchanger for heating (Nautilus AA-45 Aquahot Automatic). The experimental units used were black polypropylene boxes with rectangular shape. Each box had a capacity of 140 L and internal dimensions of 71 x 51 x 39 cm. A total of 90 L of water was filled into each box.

During the experimental period, the fish were fed four times a day *ad libitum* with commercial extruded feed [crude protein (38%), moisture content (10%), ethereal extract (7.5%), fiber (5%), calcium (3%), phosphorus (1.45%)]. Maintenance procedures were carried out throughout the experiment. Once a day, the waste of feed and feces was siphoned from the boxes. Additionally, the backwash system of the biological filters was activated four times a day for cleaning purposes. Water flow and oxygenation were regulated using taps and porous stones in all boxes. The fish were kept under natural photoperiod conditions, with light periods ranging from 11 to 13 hours over the course of the experiment.

### Physical and chemical parameters of water

The water quality parameters were measured for the five boxes of each condition. For this, a HI98311 conductivity meter (Hanna Instruments – accuracy of 0.15 ºC for temperature and 0.1µS/cm for conductivity) to measure temperature (twice a day) and conductivity (weekly), the HI98172 pH meter (Hanna Instrument - Accuracy: ± 0.1 pH) for pH (weekly), the HI9146-10 oximeter (Hanna Instruments – Resolution: 0.01 mg/L) was used to measure dissolved oxygen (daily).

### Biometrics and histological preparations

To determine the beginning of gonadal differentiation and investigate the influence of temperature on this process, five fish from each treatment (one per replicate) were randomly sampled at 50, 70, 90, 110, 130, 150, 170, 190, 210 and 240 DAE. Biometric measurements were performed determining the individual body mass in grams (BM) and standard length in centimeters (SL). After biometry, fish were euthanized with a lethal dose of benzocaine (500 mg/L). Gonads were carefully removed and fixed in modified Karnovsky's solution (4% paraformaldehyde and 2% glutaraldehyde in Sorensen phosphate buffer - pH 7.2). Fixed ovary samples were processed, included in historesin (Leica historesin embedding kit, Leica Microsystems, Nussloch, DE), sectioned at 3 µm, fixed on slides, and stained with hematoxylin-eosin. After this process, the histological sections were observed under a Leica DM4000 binocular microscope (Leica Microsystems, Wetzlar, Germany), equipped with a Leica DFC310 FX camera. Image captures were performed using Leica LAS v4.3.0 software (Leica Microsystems).

### Gonadal sexual differentiation

To ensure unbiased analysis, a blind method was adopted for evaluating sexual differentiation in this study. A single observer, who was unaware of the experimental conditions, evaluated each gonad. To characterize the sex of the individuals, specific criteria were adopted. In males, the presence of spermatogonial and spermatocyte cysts was considered indicative of male sexual differentiation. On the other hand, in females, the presence of oocytes in the primary growth stage was used as a criterion for identifying female sexual differentiation. Undifferentiated gonads were categorized into three groups based on a visual criterion for assessing the amount and type of distribution (single, in pairs and/or in cords) of primordial germ cells within the gonads. The distribution of these cells, whether single, in pairs, or in cords, has been observed to be associated with sexual differentiation in certain species (Martínez et al., 2014; Fernandino and Hattori, 2019; Ye et al., 2019). In males, the first clear sign of testicular differentiation is the appearance of the anlagen of the efferent duct; also, male germ cells are arrested in mitosis, while in females, they enter meiosis (Martínez et al., 2014).

### Statistical analysis

The statistical analysis and graph design were performed using RStudio (RStudio: Integrated Development Environment for R. Posit Software, PBC, Boston, MA. http://www.posit.co/). Assumptions such as normality and homoscedasticity were determined using the Shapiro-Wilk test and Levene’s test, respectively. Standard length and body mass over time were analyzed using a generalized linear model (GLM) with the following equation: glm(variable ~ temperature * DAE, family = Gamma(link = “inverse”)) to test the effect of temperature, time, and the interaction between temperature and time on biometric variables. Model fit was assessed by the distribution of model residuals by both histogram and Shapiro-Wilk test and by the lowest AIC value and highest logLik value. Differences were assessed by contrasts using Least-Squares Means using the Bonferroni method for p-value adjustment. To evaluate whether the sex differentiation proportions were similar in the two temperatures, a contingency table was analyzed by Fisher’s Exact Test for count data. Since we only observed both sexes differentiated at 29 °C, we evaluated potential differences in growth between females and males at 29 °C at the end of the experiment (240 DAE) using T-test and Wilcoxon test for BM and SL, respectively. All tests were performed at a significance level of *P* = 0.05, and the data were expressed as mean followed by standard error (mean ± SE).

## Results

### Water quality parameters

The mean ± SE values of the water quality parameters in the condition at 25 ºC of water temperature, pH, dissolved oxygen concentration, and water conductivity were respectively 25.0 ± 0.2 ºC, 7.3 ± 0.2, 7.8 ± 0.2 mg/L, and 101.7 ± 4.6 µS.cm. In the condition at 29 °C the mean ± SE of water temperature, pH, dissolved oxygen concentration, and water conductivity were respectively 29.0 ± 0.2 °C, 7.7 ± 0.2, 7.3 ± 0.2 mg/L, and 108.3 ± 4.3 µS.cm.

### Survival and biometric data

**Figure 1:**
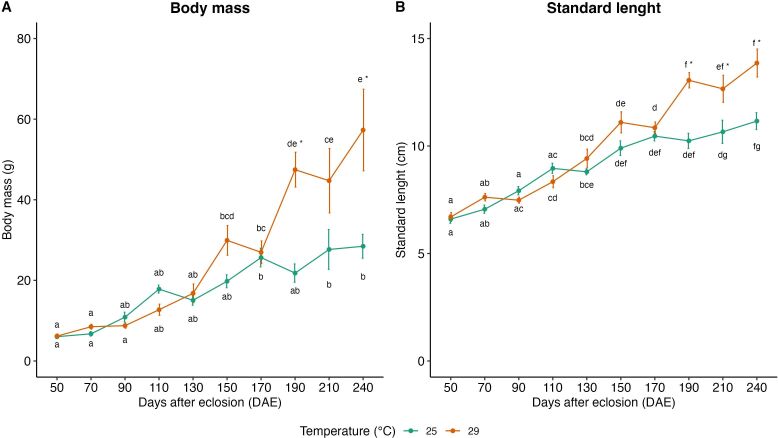
Mean ± SE values of body mass (A) and standard length (B) of *Leporinus friderici* kept in two different conditions (25 °C and 29 °C). Asterisks indicate significantly differences between conditions at the same DAE and different lower-case letters indicate significantly differences at the same condition over time (p<0.05).

Throughout the experimental period, no mortalities were observed in both temperature conditions. At the beginning of the experiment, at 50 DAE, the mean ± SE values for BM and SL were respectively 6.0 ± 0.4 g and 6.6 ± 0.2 cm, in fish kept at 25 °C, and 6.2 ± 0.5 g and 6.7 ± 0.2 cm, those kept at 29 ºC (Figure 1A, B).

The distribution profiles of BM and SL were similar in both temperature conditions up to 170 DAE. However, at 190 DAE higher biometric values were observed in the group kept at 29 ºC. The mean BM at 190 DAE was 47.5 ± 4.32 g, and the mean SL was 13.1 ± 0.35 cm at 29 °C, compared to 21.8 ± 2.27 g and 10.2 ± 0.35 cm, respectively, at 25 °C (p < 0.05). After 190 DAE, the mean values for BM and SL remained higher at 29 °C compared to 25 °C (p < 0.05) (Figure1). At the end of the experimental period, the biometric values were higher in the temperature of 29 °C (BM: 57.3 ± 10.12 g and SL: 13.9 ± 0.65 cm) compared to 25 °C (BM: 28.5 ± 2.95 g and SL: 11.2 ± 0.39 cm) (p < 0.05) (Figure 1). Additionally, the total biomass at the end of the experiment was twice as high in the 29 °C condition compared to the 25 °C condition (Figure 1). Furthermore, when comparing the biometric data of male and female differentiated animals at 29 °C in the final sampling (240 DAE), no significant difference was observed (Table 1).

**Table 1:** Relationship of the number of individuals by gender (undifferentiated, females, and males), body mass (BM), and standard length (SL) of animals collected from 50 to 240 days after eclosion (DAE) at 25 °C and 29 °C. Data are shown as mean ± standard error.

**DAE**	**Gender**	**Temperature 25 ºC**			**Temperature 29 ºC**		
		**n**	**BM (g)**	**SL (cm)**	**n**	**BM (g)**	**SL (cm)**
50	Undiff	5	6.0 ± 0.4	6.6 ± 0.2	5	6.2 ± 0.5	6.7 ± 0.2
	Females	0	-	-	0	-	-
	Males	0	-	-	0	-	-
70	Undiff	5	6.7 ± 0.6	7.1 ± 0.2	5	8.5 ± 0.6	7.6 ± 0.1
	Females	0	-	-	0	-	-
	Males	0	-	-	0	-	-
90	Undiff	5	10.9 ± 1.2	7.9 ± 0.3	5	8.8 ± 0.6	7.5 ± 0.3
	Females	0	-	-	0	-	-
	Males	0	-	-	0	-	-
110	Undiff	5	17.8 ± 1.0	9.0 ± 0.2	5	12.7 ± 1.4	8.3 ± 0.3
	Females	0	-	-	0	-	-
	Males	0	-	-	0	-	-
130	Undiff	5	15.1 ± 1.2	8.8 ± 0.1	5	16.8 ± 2.3	9.4 ± 0.4
	Females	0	-	-	0	-	-
	Males	0	-	-	0	-	-
150	Undiff	4	20.6 ± 1.8	12.1 ± 0.4	5	29.9 ± 3.7	11.1 ± 0.5
	Females	1	16.4 ± 0.0	11.4 ± 0.0	0	-	-
	Males	0	-	-	0	-	-
170	Undiff	5	25.7 ± 2.3	10.5 ± 0.2	3	23.9 ± 0.7	10.7 ± 0.2
	Females	0	-	-	2	27.6 ± 8.5	10.7 ± 0.7
	Males	0	-	-	0	-	-
190	Undiff	5	21.8 ± 2.3	10.2 ± 0.4	0		
	Females	0	-	-	4	49.8 ± 4.7	13.3 ± 0.3
	Males	0	-	-	1	38.0 ± 0.0	12.1 ± 0.0
210	Undiff	5	27.7 ± 5.0	10.7 ± 0.5	0		
	Females	0	-	-	3	42.5 ± 13.5	12.4 ± 1.0
	Males	0	-	-	2	48.0 ± 8.6	13.1 ± 0.6
240	Undiff	3	26.2 ± 1.3	11.0 ± 0.2	0		
	Females	2	31.8 ± 8.0	11.4 ± 1.2	3	63.1 ± 17.3^a^	13.9 ± 1.2^a^
	Males	0	-	-	2	48.6 ± 1.6^a^	13.9 ± 0.1^a^
**Mean**	**Undiff**	**47**	**17.4 ± 1.3**	**9.3 ± 0.3**	**33**	**14.7 ± 1.6**	**9.0 ± 0.4**
	**Females**	**3**	**26.6 ± 6.9**	**11.4 ± 0.7**	**12**	**47.6 ± 6.1**	**12.8 ± 0.5**
	**Males**	**0**	**-**	**-**	**5**	**46.3 ± 3.4**	**13.2 ± 0.4**

DAE: days after eclosion; n: sample size; BM: body mass; SL: standard length; Undiff: undifferentiated; shaded in gray - onset of differentiation in females; shaded in blue - onset of differentiation in males. The same superscript letters at 240 DAE indicated that there were not significantly differences in BM and SL between males and females (p > 0.05).

### Gonad differentiation

**Figure 2:**
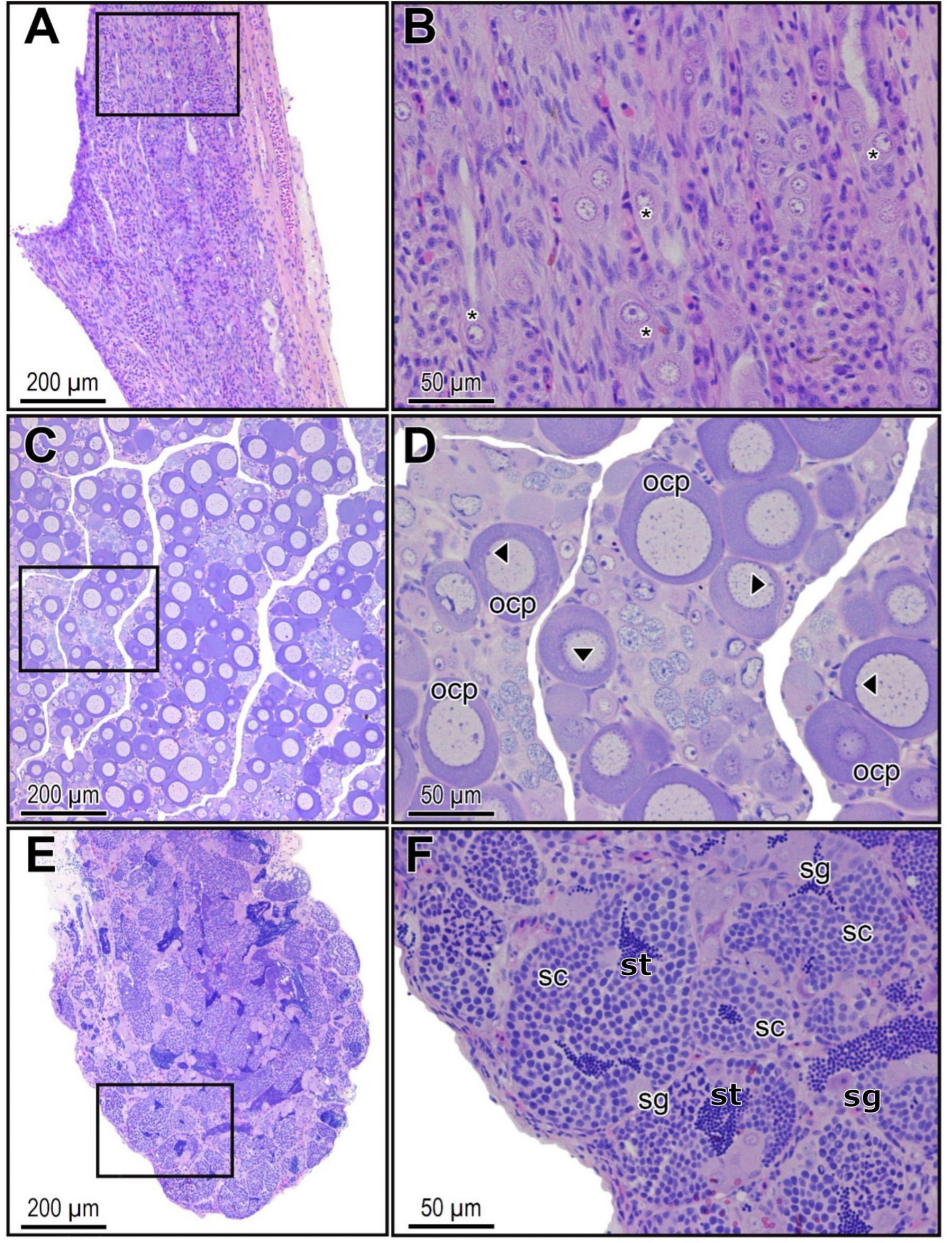
Photomicrograph of the longitudinal section of the gonadal tissue of *Leporinus friderici*. (A) and (B) undifferentiated gonad at 150 days after hatching; (C) and (D) differentiated ovary in an animal kept at a controlled temperature of 29 ºC; (E) and (F) differentiated testis in an animal kept at a controlled temperature of 29 ºC. Figures “E” and “F” show that most tubules cross section contain spermatozoa in the center of seminiferous tubules. Highlight: square – delimitation of the enlarged region on the right side; asterisks: primordial germ cell; arrowhead: nucleoli; ocp: primary growing oocytes; sg: spermatogonia; sc: spermatocytes; st: sperm. Staining with HE.

From 50 DAE to 150 DAE, only primordial germ cells (PGCs) and somatic cells were observed, along the longitudinal axis of the undifferentiated gonads, in both temperatures. During this period, the PGCs remained quiescent, and the gonads remained undifferentiated in both temperatures (Figure 2A, B). PGCs were large cells presenting a large nucleus with loose chromatin and prominent nucleoli. PGCs were surrounded numerous smaller and flattened somatic cells (Figure 2B). From 150 DAE onwards, ovaries were observed, evidenced by numerous oocytes in primary growth (Figure 2C, D). Finally, from 190 DAE, only at 29 ºC, testes were observed, evidenced by spermatogonia, spermatocytes, spermatids and lumen filled with spermatozoa (Figure 2E, F).

**Figure 3:**
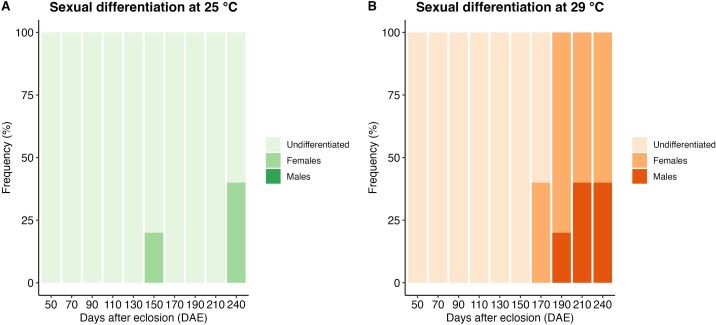
Frequency of sexual differentiation of *Leporinus friderici* kept at 25 °C (A) or 29 °C (B**)** from 50 to 240 days after hatching (DAE). n= 5 animals per treatment at each sampling.

At 25 °C, the differentiation process occurred earlier at 150 DAE, but it was less intense, with only three animals showing differentiation throughout the entire experimental period. In contrast, at 29 °C, 17 animals showed differentiation (Figure 3). The absence of individuals differentiated into males at 25 ºC prevented a statistical analysis of the sex ratio for comparison between conditions. However, considering all the observed animals throughout the experimental period, the sex ratio at 25 °C was 6% females, 0% males, and 94% undifferentiated. On the other hand, at 29 °C, the sex ratio was 34% females, 10% males, and 66% undifferentiated. There was a significant difference between the sex differentiation proportions in the two temperatures (p < 0.05). This finding suggests that at 29 °C, the sex differentiation process was intensified compared to the process observed at 25 °C (Table 2).

**Table 2:** Contingency **table of** sex differentiation of *Leporinus friderici* kept in two different conditions (25 °C and 29 °C) from 50 to 240 days after eclosion (DAE).

**Temperature**	**Sex differentiation**			**TOTAL**
	**Females**	**Males**	**Undifferentiated**	
25 °C	3	0	47	50
29 °C	12	5	33	50
TOTAL	15	5	80	100

## Discussion

In this study, it was evident that the elevated temperature of 29 °C intensifies the sex differentiation process in *L. friderici* individuals, resulting in a greater number of individuals differentiating compared to those at 25 °C (17 individuals at 29 °C and only three individuals at 25 °C). Moreover, by 190 DAE (140 days after the start of the experiment), the differences in BM and SL become significant, and the animals (including those undifferentiated) maintained at 29 °C were approximately 1.27 times larger and had 2.18 times more mass compared to those maintained at 25 °C. Unfortunately, due to the limited number of males differentiating at both temperatures, a statistical evaluation of the sex ratio based on the two temperatures (25 °C and 29 °C) could not be performed. Additionally, the small number of differentiated males prevented conclusive comparisons of body mass and standard length between male and female individuals at 25 °C.

The process of transitioning from undifferentiated gonads to ovaries (150 DAE) occurred earlier compared to the differentiation to testes (190 DAE). The observed pattern of differentiation observed in this study, where differentiation into ovaries occurs before differentiation into testes, is consistent with what has been commonly described in the majority of Neotropical fish species such as piracanjuba (*Brycon orbignyanus*) (Zardo et al., 2021; Quirino et al., 2022), pacu (*Piaractus mesopotamicus*) (Barbosa et al., 2022), lambari (*Astyanax altiparanae*) (Adolfi et al., 2015) and pirarucu (*Arapaima gigas*) (Amaral et al., 2020). The differentiation process was initiated at 150 DAE at 25 °C and at 170 DAE at 29 °C. However, despite initiating 20 days earlier at the lower temperature, only three individuals (all females) had differentiated by the end of the experiment at 25 °C. In contrast, at 29 °C, a total of 17 individuals differentiated (12 females and five males).

Therefore, we can conclude that the higher temperature of 29 °C intensified the differentiation process, supported by the fact that males were only identified at this temperature. Another crucial factor for this conclusion was that animals with undifferentiated gonads were observed throughout the entire experimental period at 25 °C, whereas after differentiation began at 170 DAE at 29 °C, no more undifferentiated animals were observed in this group. The significant difference in sex differentiation proportions between the two temperatures further supports the conclusion that the elevated temperature of 29 °C has a significant impact on the sex differentiation process in *L. friderici*. Additionally, the intensification of sex differentiation at higher temperature seems to be more related to fish size than the age, as previously demonstrated in other teleosts (Gao et al., 2009). After differentiation onset, the body mass and standard length of animals maintained at 25 °C were kept constant along the experimental period unlike the observed at 29 °C, which presented a gradual increase in these values from 170 DAE. This difference in growth rate may also have been reflected in the differentiation of individuals into males, since individuals at 25 °C did not reach the minimum size of males differentiated at 29 °C (12.1 cm) throughout the experimental timeframe.

In this context, temperature is known to be one of the main environmental factors effecting on the processes of sexual differentiation, gametogenesis and even spawning in teleosts. Temperature-dependent sex determination (TSD) is widely studied, either as an alternative for monosex production in aquaculture or the context of climate change. It is now well accepted that TSD exists in fish (Honeycutt et al., 2019) and other aquatic animals such as turtles (Hawkes et al., 2007; Santidrián Tomillo and Spotila, 2020). In addition to the effect on sexual differentiation, higher-than-optimal temperatures can advance, delay or even inhibit oocyte development and maturation as well as spawning in several species (Alix et al., 2015; Lema et al., 2024).

In this study, we did not observe significant differences in terms of BM and SL between male and female analyzed in the 29 °C condition at 240 DAE (i.e., the end of the experimental period). Therefore, even though it is known that *L. friderici* females reach a larger size than males in their natural environment (Lopes et al., 2000; Rêgo et al., 2008), under the conditions used in this study and the experimental timeframe, we did not observe differences in this parameter at the end of the experiment among the analyzed animals. This finding raises some reflections on the allocation of energy between gonadal and somatic growth and maturation. It is accepted that when animals allocate energy to the gonads at the time of first maturation or puberty (i.e., when they start gamete production), it is diverted from somatic growth to gonadal growth, resulting in a reduction in growth rate (Kissil et al., 2001; Imsland et al., 2012). In this study, we observed that differentiated females had ovaries with oocytes in the stationary phase of primary growth without initiating the vitellogenic process. It is known that vitellogenesis is an energy-demanding process, mainly due to vitellogenin synthesis (Reading et al., 2018), and it is dependent on the activation of the brain-pituitary-gonad (BPG) axis and the stimulation of estradiol production through FSH stimulation, which is crucial for the initiation of this process (Senthilkumaran et al., 2004). Therefore, subjecting the animals to elevated temperatures seems to have intensified the differentiation process without leading to early vitellogenesis in females.

Thus, at least in females, it is not possible to determine if the BPG axis (Servili et al., 2020) has been activated due to the elevated temperature. Although we did not measure the plasma concentration of substances involved in this BPG axis cascade, we observed that the ovaries of females maintained at different temperatures were morphologically similar and only contained oocytes in the primary growth phase without initiating the vitellogenic process. Therefore, these differentiated females can be classified as pre-pubertal (Taranger et al., 2010) since they have not yet undergone the process of first gonadal maturation under the conditions established in our study. Conversely, in the present study, we found haploid cells in recently undifferentiated males, indicating that in males, the differentiation into testes is followed by the initiation of first maturation with the production of male gametes and the subsequent closure of the Sertoli cells barrier (Leal et al., 2009). Analyzing the plasma concentrations of testosterone and estradiol during differentiation would be interesting in future approaches to help elucidate the mechanisms associated with differences in biomass between temperatures without apparent interference from differentiation in this phenomenon.

In this concern, an early differentiation of females, but without vitellogenesis and a later differentiation of males, but with an immediate entry into the initial maturation and puberty process seems to explain the similar biometric data between genders during the experimental period. In other words, *L. friderici* females differentiate earlier but seems not to invest energy in vitellogenesis immediately after that, but the time between female differentiation and female first maturation needs to be further elucidated. In this aspect, a longer period of analysis would be necessary to identify any differences in growth rates between males and females that could justify the production of a monosex population. Intense differences in biometric data among specimens of same age before differentiation were found in another native migratory fish, *P. mesopotamicus* (Barbosa et al., 2022). So, it seems that growing rates are not necessarily related to sex differentiation in some migratory tropical fish used in aquaculture. Thus, unlike tilapia, where the growth differences between males and females are prominent before the harvest weight (reviewed in Taranger et al., 2010), for medium to large-sized migratory characiform fish such as *P.mesopotamicus* (Barbosa et al., 2022) and *L.friderici* (present study) this issue remains to be elucidated.

**Figure 4:**
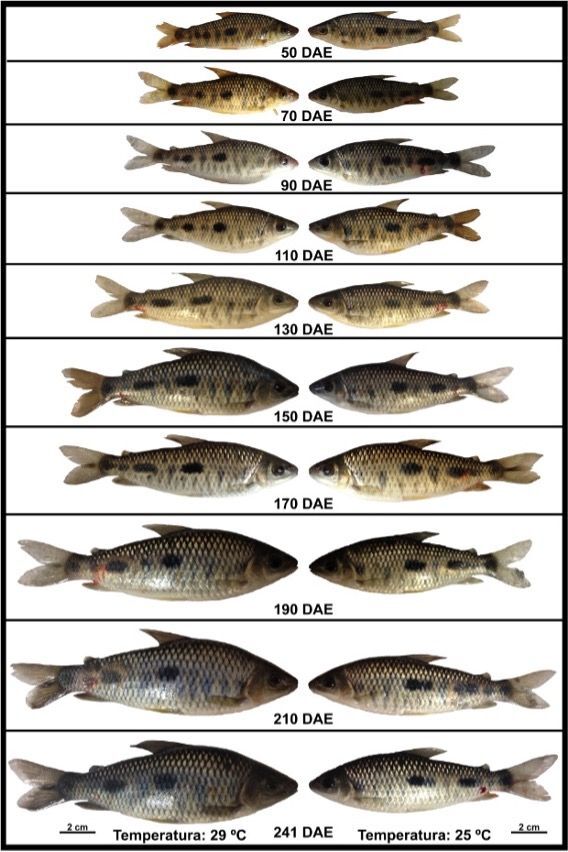
The figure shows the evolution of size and mass of *Leporinus friderici* specimens kept at different temperatures.

**Figure 5:**
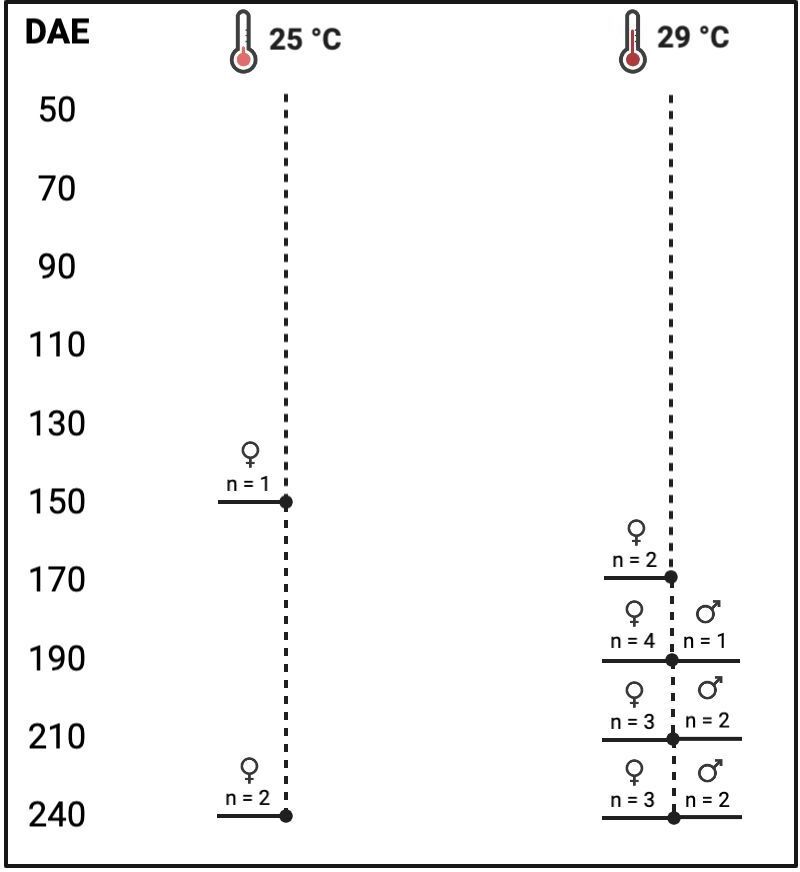
The figure shows the different intensities in the *Leporinus friderici* differentiation processes at different temperatures.

While it was not possible to measure the effect of temperature on the sex ratio, high temperature proved to be a decisive factor for the increase in body mass in animals kept at 29 °C. The final body mass obtained at 29 °C, irrespective of the differentiation process, was double that obtained at the lower temperature, reaching 57.3 ± 10.12 g and 13.9 ± 0.65 cm, in contrast to 28.5 ± 2.95 g and 11.2 ± 0.39 cm at the lower temperature. Little is known about the harvest size of *L.friderici* in production systems, as most of the information comes from average weights obtained through fishing, ranging from 102.5 g for males to 164.23 g for females (Rêgo et al., 2008). The implications of the arbitrary use of temperature on differentiation could lead, for instances, the release of populations with different degrees of proportion of differentiation than that occurring in natural habitats in fish restocking programs (Figures 4 and 5). In these programs, thousands of individuals are maintained until they reach a size between 10 and 15 cm. This aspect may be considered for the release of smaller and younger individuals, allowing differentiation to occur according to the temperature of the environment where they are being released, without altering the biology of the species. Another point that could be explored is that, since an increase in temperature resulted in an intensification of the sexual differentiation process, we cannot rule out the possibility that temperature has directly affected sex determination or the genetic mechanisms of sex determination in this species (reviewed in Lema et al., 2024). In this way, the wild population of *L. friderici* could be affected by the current rapid climate change, resulting in alterations concerning differentiation process that could have unpredictable ecological consequences, especially on population recruitment in response to increases in water temperature.

## Conclusion

In this study, we concluded that the higher temperature (i.e., 29 ºC) intensified the gonadal differentiation process of *L. friderici* and provided higher mean body mass and standard length than 25 °C. Maintaining fish at 29 °C during the differentiation process can be an alternative to intensify the gonadal differentiation process as well as to accelerate growth in this species as no mortality was observed. It can also be an important tool for fish stocking programs to fastest obtain the minimum size required for fish release.
